# AIO LQ-0110: a randomized phase II trial comparing oral doxycycline versus local administration of erythromycin as preemptive treatment strategies of panitumumab-mediated skin toxicity in patients with metastatic colorectal cancer

**DOI:** 10.18632/oncotarget.21249

**Published:** 2017-09-23

**Authors:** Melanie Kripp, Nicole Prasnikar, Ursula Vehling-Kaiser, Julia Quidde, Salah-Eddin Al-Batran, Alexander Stein, Kai Neben, Carla Verena Hannig, Hans Werner Tessen, Tanja Trarbach, Axel Hinke, Ralf-Dieter Hofheinz

**Affiliations:** ^1^ Medizinische Klinik 3, Hämatologie und Onkologie, Universitätsmedizin Mannheim, Mannheim, Germany; ^2^ Hämatologie, Internistische Onkologie und Palliativmedizin, Asklepios Klinik Barmbek, Hamburg, Germany; ^3^ Tagesklinik für Hämatologie, Onkologie, Palliativmedizin, Landshut, Germany; ^4^ Universitäres Cancer Center, UKE Hamburg, Hamburg, Germany; ^5^ Universitäres Centrum für Tumorerkrankungen, Krankenhaus Nordwest, Frankfurt, Germany; ^6^ Medizinische Klinik II, Klinikum Mittelbaden, Baden-Baden, Germany; ^7^ Onkologisches Gemeinschaftspraxis, Bottrop/Dorsten, Germany; ^8^ Onkologische Kooperation Harz, Goslar, Germany; ^9^ Zentrum für Tumorbiologie und Integrative Medizin, Klinikum Wilhelmshaven, Wilhelmshaven, Germany; ^10^ WiSP Wissenschaftlicher Service Pharma GmbH, Langenfeld, Germany; ^11^ Interdisziplinäres Tumorzentrum, Universitätsmedizin Mannheim, Universität Heidelberg, Heidelberg, Germany

**Keywords:** doxycycline, erythromycin, panitumumab, skin toxicity, WoMo score

## Abstract

**Background:**

Dermatologic toxicities, especially akne-like skin rash, are the most common side-effects associated with anti-epidermal growth factor receptor (EGFR) therapy. Preemptive treatment with oral tetracyclines is recommended as a standard. Topical prophylactic options have thus far not been compared to tetracyclines. In the current study, we sought to establish an alternative topical treatment.

**Patients and methods:**

In this multicentre, randomized, open-label phase II study patients with (K)Ras-wildtype colorectal cancer receiving panitumumab were randomized (1:1) to receive either doxycycline 100 mg b.i.d. (standard arm) or erythromycin ointment 2% followed by doxycycline in case of insufficient activity. The primary endpoint was the percentage of patients developing no skin toxicity ≥ grade 2 at any time during the first 8 weeks of panitumumab treatment. Skin toxicity was assessed using the NCI CTCAE v 4.0. Secondary endpoints comprised the assessment of skin toxicity using a more thorough grading system (WoMo score), evaluation of skin-related (DLQI) and global quality of life (EORTC QLQ C30).

**Results:**

In total, 88 patients were included in this trial. 69% of the patients in the erythromycin arm suffered from skin toxicity of grade ≥ 2 versus 63% in the standard arm (*P = n.s*.). However, as per WoMo score significantly more patients in the erythromycin arm developed moderate or severe skin toxicity at earlier time points. Skin related and overall quality of life was comparable between both arms.

**Conclusions:**

Based on this data erythromycin cannot be regarded as an alternative to doxycycline as prevention of EGFR-related skin toxicity.

## INTRODUCTION

Dermatologic toxicities are the most common side-effects associated with anti-EGFR therapy and they are experienced by the majority of patients undergoing EGFR inhibitor therapy. While toxicities are mild (grade 0-1) to moderate (grade 2) in most patients, about 15-20% exhibit higher grade toxicities leading to medical and psychosocial effects that may result in poor patients’ compliance with lower adherence to cancer therapy, more dose delays, and interruption or discontinuation of antineoplastic therapy [1, 2]. Finally, dermatologic toxicities may contribute to a significantly reduced quality of life [2, 3, 4]. Acneiform rash, usually developing within the first 2 to 4 weeks of therapy, is by far the most frequent type of skin toxicity caused by EGFR antibody treatment.

Management guidelines for the prophylaxis and treatment of EGFR inhibitor-mediated dermatologic toxicities usually recommend prophylactic use of tetracyclines and special skin care. Indeed, these recommendations are based on few randomized trials:

Jatoi et al. randomized 62 patients treated with anti-EGFR antibodies or tyrosine kinase inhibitors (TKIs) between tetracycline and placebo. While the overall incidence of skin toxicity was not reduced, a significant decrease of severity was observed. Of note, patients treated with tetracycline reported improved skin-related quality of life as assessed by the SKINDEX-16 quality of life (QoL) questionnaire [5]. In a subsequent similar study including 65 patients Jatoi and coworkers could not confirm these findings [6]. Scope et al. randomized 48 colorectal cancer patients treated with cetuximab between minocycline and placebo. Minocycline significantly decreased moderate to severe itch. Likewise, a trend toward a decreased incidence of moderate to severe rash was noticed (20% vs. 42%, *P* =.*13*) [7]. Deplanque et al. randomized 147 patients with non-small-cell lung cancer (NSCLC) between treatment with erlotinib alone or in combination with doxycycline. Doxycycline did not reduce the incidence of folliculitis over untreated patients (71% vs. 81%, *P* =.*117*) but significantly reduced its severity (*P* =.*001*) [8]. Melosky et al. randomized 150 patients with NSCLC treated with erlotinib between prophylactic minocycline, reactive minocycline and no treatment. The incidence of skin toxicity was 84% regardless of treatment arm. Prophylactic treatment with minocycline, however, significantly lengthened the time to the most severe grade of rash. Grade 3 rash was significantly higher in the no-treatment arm [9]. Arrieta et al. randomized 90 patients with NSCLC treated with afatinib between preemptive tetracycline and only reactive treatment. Tetracycline reduced the rash incidence of any grade (44.5% vs. 75.6%, *P* =.*046*) and the severity of grade ≥ 2 (15.6% vs. 35.6%, *P* =.*030*) over reactive treatment [10]. Finally, Lacouture et al. randomized 114 patients with NSCLC treated with dacomitinib between preemptive doxycycline and placebo. Doxycycline significantly reduced the incidence of select dermatologic adverse events of interest (SDAEI) grade ≥ 2 by 50% (*P* =.*016*) [11].

In all, it appears to be justified to recommend a prophylaxis with tetracyclines such as doxycycline or minocycline. Two randomized studies confirmed that tetracyclines should be used as prophylaxis and not as an early intervention: Firstly, the so-called STEPP trial randomized 95 patients with metastatic colorectal cancer (mCRC) to be treated with panitumumab in combination with chemotherapy to either preemptive treatment for skin toxicity including doxycycline 100 mg b.i.d. or to receive the same regimen reactively. Incidence of skin toxicity grade ≥ 2 was significantly reduced by preemptive treatment compared with reactive treatment (29% vs. 62%). Moreover, patients randomized to preemptive treatment reported improved QoL according to Dermatology Life Quality Index (DLQI) especially between week 2 and 3 [3]. Secondly, the so-called J-STEPP trial confirmed these data using a comparable study design in a Japanese population with mCRC. Comparable to the STEPP trial, the incidence of ≥ grade 2 skin reactions was decreased with preemptive treatment compared with reactive treatment (21.3% vs. 62.5%, *P* <.*001*) [12].

Thus far, no data comparing oral antibiotics with a local treatment have been reported. In order to avoid a long term exposure to oral antibiotics a preemptive topical skin treatment could be useful. Moreover, topical agents are more convenient than oral antibiotics, and compliance might be better. In the present trial we sought to compare a local treatment using erythromycin followed by doxycycline in the case of insufficient activity with doxycycline. Erythromycin seemed to be very suitable as it belongs to standard treatment for acne vulgaris [13].

## RESULTS

### Patients

In total, 88 patients were randomized by 11 sites between July 2011 and October 2014. 80 out of 88 patients were evaluated (41 patients in the doxycycline arm, 39 patients in the erythromycin arm). Reasons for exclusion were: withdrawal of consent, no therapy started (n = 4), no post-baseline skin toxicity assessment (n = 1); treatment not started due to RAS mutation (n = 2), investigator´s decision (n = 1).

Patients´ and major tumor-related baseline characteristics are depicted in Table 1.

**Table 1:** Major patients´ and tumour characteristics

Parameter	Doxycycline	Erythromycin	Total
Age			
*N*	40^*^	*39*	*79*
Mean ± SD (years)	68.1 ± 12.4	69.1 ± 9.4	68.6 ± 11
Median (years)	69.5	70.3	70.2
**Gender**			
*N*	*41*	*39*	*80*
Female	13 (32%)	18 (46%)	31 (39%)
Male	28 (68%)	21 (54%)	49 (61%)
**Cancer location**			
*N*	*41*	38^*^	*79*
Colon	23 (56%)	18 (47%)	41 (52%)
Rectum	18 (44%)	20 (53%)	38 (48%)
**Performance status**			
*N*	39^*^	38^*^	*77*
ECOG 0	16 (41%)	17 (45%)	33 (43%)
ECOG 1	19 (49%)	19 (50%)	38 (49%)
ECOG 2	4 (10%)	2 (5%)	6 (8%)

^*^Data missing.

### Primary end point

The percentage of patients developing skin toxicity according to NCI CTC criteria is shown in Table 2. In the erythromycin arm, 69% suffered from skin toxicity grade ≥ 2 (95% confidence interval [CI]: 52% to 83%; primary endpoint). In the standard arm with doxycycline the toxicity rate with grade ≥ 2 amounted to 63% (95% CI: 47 – 78%). Thus, the primary endpoint was not met.

**Table 2:** Skin toxicity graded according to NCI CTCAE 4.0, worst per patient

NCI CTCAE grade	Doxycycline	Erythromycin	Total
*N*	*41*	*39*	*80*
0 / no toxicity	3 (7%)	3 (8%)	6 (8%)
1	12 (29%)	9 (23%)	21 (26%)
2	18 (44%)	12 (31%)	30 (38%)
3	8 (20%)	15 (38%)	23 (29%)

*P* =.*27* (Mantel-Haenszel test for trend, exact version).

Although this randomized phase II study was not powered for formal statistical comparisons of the two treatment groups, results from additional statistical calculations are provided in a descriptive way: Using Fisher's exact test no significant differences between both arms were seen (*P* =.*64)*. The odds ratio, with values > 1.0 indicating higher toxicity, was 1.29 (95% confidence interval: 0.46 – 3.67; one-sided 95% confidence limit for inferiority of the erythromycin arm: 3.15). In absolute values, the rate difference of 6% more toxicity in the erythromycin group has a 95% confidence interval (according to HAUCK-ANDERSON) ranging from −16% to + 28%.

**Figure 1:**
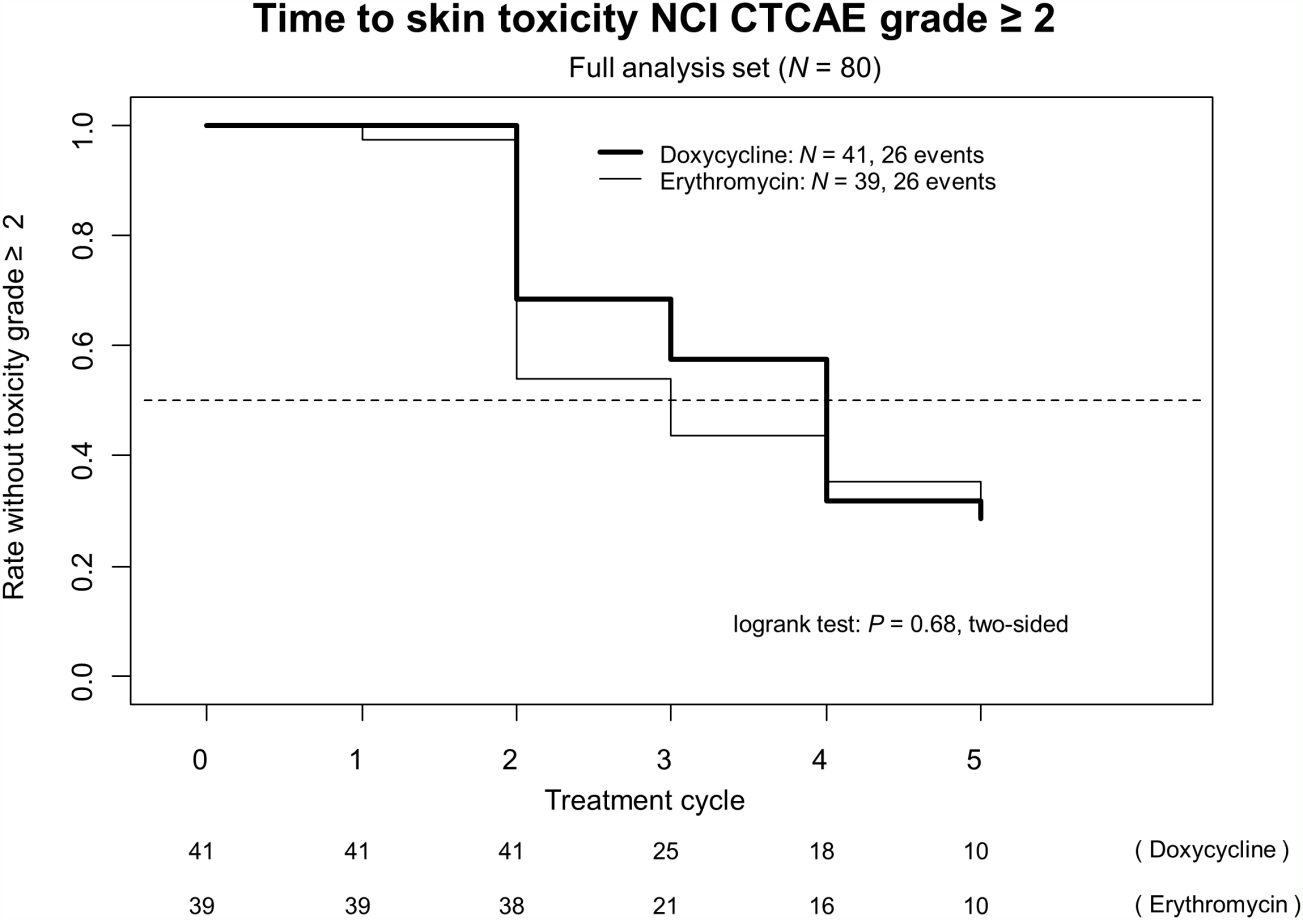
Time to skin toxicity NCI CTCAE grade ≥ 2 (full analysis set [N = 80])

Figure 1 shows the time to the onset of skin toxicity of NCI CTCAE grade ≥ 2. In the doxycycline arm median time was 43 days (day one of cycle 4), in the erythromycin arm median time was 29 days (day one of cycle 3) without statistical significance in the corresponding logrank test (*P* =.*68*).

### Skin toxicity graded according to WoMo score

The total score result from the WoMo assessment over time (individual cycles) show a constantly higher score in the erythromycin group (data not shown). When the maximum WoMo score by patient over time is analyzed (Table 3), the difference amounts to almost 10 points which is about half of the standard deviation.

**Table 3:** Skin toxicity according to WoMo score

WoMo score	Doxycycline	Erythromycin	Total
*N*	*41*	*39*	*80*
Mean ± SD	25.6 ± 16.7	34.1 ± 19.8	29.7 ± 18.7
Median	23.1	32.1	29.5
Quartiles	14.5 - 34.8	21.9 - 47.2	16.1 - 40.9
Range	0 - 68.2	0 - 88.1	0 - 88.1

A significantly higher number of patients treated in the erythromycin arm developed moderate or severe skin toxicity according to WoMo score, i.e. ≥ 20 points (30/39 versus 23/41 patients, *P* = *.049*); moreover, a higher number of patients had severe skin toxicity (WoMo score ≥ 40 points 14/39 versus 8/41 patients; *P* =.*101*).

**Figure 2:**
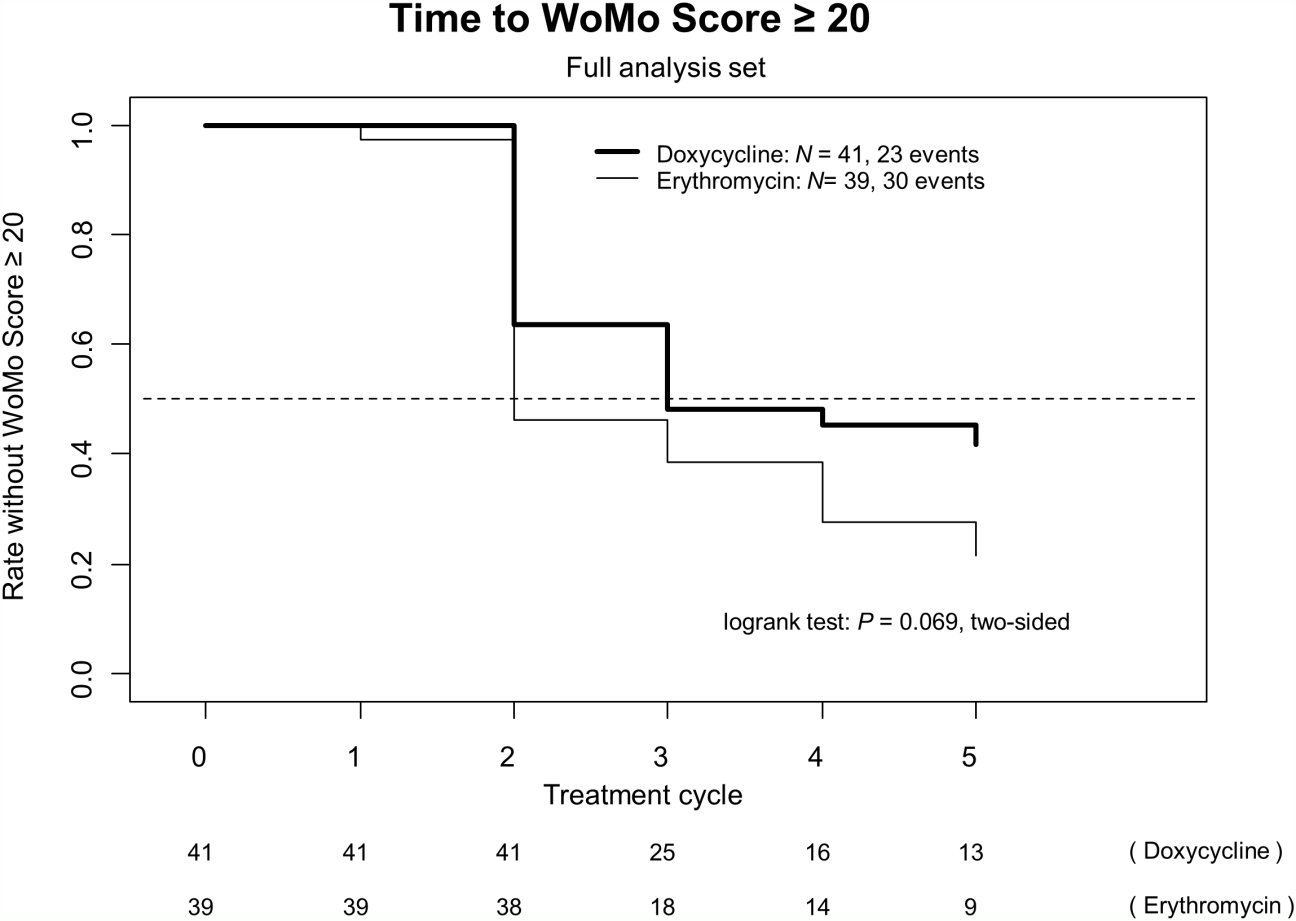
Time to WoMo score ≥ 20 (full analysis set [N = 80])

Analyzes based on worsening of the WoMo score were performed. It took one cycle longer to appearance of a moderate to severe skin toxicity (WoMo score ≥ 20) with doxycycline compared to erythromycin. Figure 2 shows the time to increase of the WoMo score to ≥ 20. An obvious difference between both arms appeared on day 15 (day one of cycle 2) and this remained discernible thereafter indicating a clear trend towards superior efficacy of the standard arm using doxycycline (*P* =.*069*).

### Quality of life

The mean values of the EORTC QLQ C30 global health score are balanced at the beginning of the first treatment cycle with about 52 points for each group (albeit based on a lower number of valid questionnaires in the erythromycin group; Table 4). Over time, the mean values amount to 59 in the erythromycin arm and decline to 47 points in the doxycycline arm (at the beginning of cycle 4). The between-group differences range between 5 and 10 points per cycle.

**Table 4:** Quality of life graded according to EORTC QLQ C30: Global health score

**Erythromycin arm**					
**Cycle**	**1**	**2**	**3**	**4**	EOT^*^
*N*	*32*	*36*	*27*	*28*	*31*
Mean ± SD	52.3 ± 23.3	53.2 ± 17.6	52.8 ± 18.5	59.2 ± 13.5	55.4 ± 17.6
Median	50	50	50	54.2	58.3
Quartiles	33.3 - 66.7	41.7 - 66.7	41.7 - 66.7	50 - 66.7	41.7 - 66.7
Range	0 - 91.7	16.7 - 83.3	0 - 83.3	33.3 - 83.3	16.7 - 91.7
**Doxycycline arm**					
**Cycle**	**1**	**2**	**3**	**4**	EOT^*^
*N*	*40*	*31*	*31*	*25*	*25*
Mean ± SD	52.9 ± 18.7	48.4 ± 17	47 ± 19.5	47.3 ± 21.2	44.7 ± 20.1
Median	50	50	50	50	50
Quartiles	33.3 - 66.7	33.3 - 66.7	33.3 - 58.3	33.3 - 66.7	33.3 - 58.3
Range	16.7 - 91.7	16.7 - 83.3	0 - 83.3	0 - 83.3	0 - 83.3

^*^End of treatment.

No relevant differences between both two treatment strategies were noticed as per the DLQI. Throughout the observation period of 8 weeks, the median values of the DLQI total score in both arms are low with a highest medium value of 4 points for both arms at the beginning of cycle 3 (except for the rise of the median at the end-of treatment assessment in the doxycycline group with a very low questionnaire compliance in this arm at that time point) (Table 5). Considering the subscales, only “symptoms and feelings” indicated major deterioration with a median of 2 points on a maximum scale of 6 (data not shown).

**Table 5:** Skin related quality of life graded according to DLQI (total score)

**Erythromycin arm**					
**Cycle**	**1**	**2**	**3**	**4**	EOT^*^
*N*	*24*	*30*	*29*	*24*	*31*
Mean ± SD	1.2 ± 2.6	5.3 ± 5.4	5.4 ± 5.6	4.4 ± 4.1	5.4 ± 5.8
Median	0	3	4	3.5	3
Quartiles	0 - 1	1.2 – 8	2 - 7	1 – 6.2	2 – 6
Range	0 - 10	0 – 25	0 - 27	0 - 14	0 – 26
**Doxycycline arm**					
**Cycle**	**1**	**2**	**3**	**4**	EOT^*^
N	24	27	23	20	15
Mean ± SD	0.8 ± 1.2	4.4 ± 4.8	5.3 ± 5.2	4.9 ± 5.4	5.8 ± 4.4
Median	0	2	4	3	7
Quartiles	0 −1	1 – 5.5	2 – 7.5	1.8 – 5.2	2 – 9
Range	0 - 5	0 - 19	0- 21	1 - 22	0- 13

^*^End of treatment.

### Treatment administration and safety

In total, 140 panitumumab administrations were recorded in the doxycycline arm (mean 3.4 per patient), and 139 in the erythromycin arm (3.6 per patient). 74% of the patients received all 4 scheduled administrations without differences in both arms. In the erythromycin arm, 33% of the patients stopped erythromycin due to insufficient activity and switched to doxycycline. Panitumumab dose reductions or dose delays were infrequent (see Supplementary Materials).

As mentioned, detailed analysis of adverse events beyond skin toxicity was not within the scope of the current study. However, neither unexpected events nor frequencies were detected within the framework of the adverse events and SAE reporting in this trial. Eleven out of 41 patients (26.8%) treated within the doxycycline arm, and 7/39 patients (17.9%) in the erythromycin arm reported SAE (*P* =.*34*).

### Antitumor efficacy

Regarding the overall study population, 28% of the patients experienced an objective response, while 46% had stabilization of the disease. This estimation is based on 54 patients (68%) with a valid RECIST evaluation after 8 weeks. There is no indication for an effect of the randomized assignment of the skin treatment (*P* =.*40*, Fisher's exact test; odds ratio: 1.74, 95% confidence interval: 0.49 – 6.67).

## DISCUSSION

This is the first randomized trial comparing an oral antibiotic prophylaxis (doxycycline) with an alternative local application (erythromycin) as preemptive skin treatment strategy against anti-EGFR targeted drugs. The primary endpoint of the present trial was the percentage of patients developing no skin toxicity grade ≥ 2 at any time during the first 8 weeks of panitumumab treatment. In the erythromycin arm, a moderate to severe skin toxicity could be prevented in only 31%, i.e. 69% suffered from skin toxicity grade ≥ 2. Thus the primary endpoint was missed and the experimental strategy (local erythromycin) would be rated as insufficient. In the reference arm, however, using doxycycline, the toxicity rate grade ≥ 2 amounted to 63%, as well, i.e. it was not lower than in the erythromycin arm. A numerical trend to a lower grade 3 toxicity with oral doxycycline can be discerned but without reaching statistical significance. Although most of the studies investigating tetracycline-family antibiotics in a preemptive setting could not demonstrate a reduction of the overall incidence of skin toxicity, they often showed a decrease of the severity compared to placebo [5, 7, 11], no treatment [8, 9], or reactive treatment [3, 9, 10, 11]. This effect could not be seen in the current trial comparing two different preemptive treatment strategies.

The rates of skin toxicity grade ≥ 2 are rather high with 69% and 63%, respectively, compared to the literature. Even if considering only trials with similar settings (mCRC treated with EGFR inhibitors) the rates of grade ≥ 2 skin toxicity in the preemptive arms are considerably lower in previous studies with 20% [5], 21.3% [12] and 29% [3]) and could not be reproduced in the present trial.

Until recently, the NCI CTCAE v 3.0 grading scale (published in 2006) had been used in most of the studies for evaluation of EGFR inhibitor induced skin toxicity [3, 5, 6, 8, 9, 12]. This version 3.0 included only a crude scale for severity of skin toxicity and allowed for subjective grading. The updated CTCAE v 4.0 used in the present trial provide a more thorough grading of skin toxicity taking into account that acneiform skin rash may develop only in lower percentage of skin surface. Patients that would have been classified grade 1 according to the NCI CTCAE v 3.0, could easily be regarded as grade 2 or even 3 according to the NCI CTCAE v 4.0. Thus, especially the results of the STEPP trial [3] on which the present trial was based cannot be compared with our data concerning the evaluation of skin rash. It is conceivable that the high amount of grade ≥ 2 toxicities in the present trial is due to the updated and improved grading system.

Nevertheless, even the updated version 4.0 does not accurately reflect the clinical situation. One major problem of this version is that the grading is based on the affected body surface area (BSA) rather than on the (local) severity/symptoms of skin rash. An upgrading beyond the grade stipulated by the grading system is principally possible (for instance due to limited instrumental or self-care activities of daily living) but this decision is left up to the discretion of the investigator. This may of course pose problems with respect to the comparability of skin toxicity between individual investigators and between studies.

In view of this, we also used an extended alternative grading system for EGFR-related skin toxicity (so called WoMo score [20]) in the present trial which takes into account also (i) the percentage of the facial area affected with rash or other dermatological adverse events as well as (ii) the type and severity of the efflorescences. Thus, the WoMo score allows for a more thorough description of the skin toxicity and enables more precise description and comparison of efficacy of different prophylactic treatments.

Notably, using this grading system, we detected obvious differences between the two treatment groups. Firstly, the mean and median WoMo scores were numerically higher in the erythromycin arm. Secondly, the percentage of patients developing moderate or severe skin rash (WoMo score ≥ 20 points) was significantly higher in the erythromycin group and the time to development of moderate or severe skin toxicity was shorter in the erythromycin group (at the beginning of cycle 2 versus cycle 3). This data indicates that the experimental stepwise approach (erythromycin followed by doxycycline) was not able to at least accomplish the same results as immediate doxycycline prophylaxis. In this regard it is important to stress that the treatment intensity and adherence to panitumumab as well as the therapeutic efficacy was comparable between both arms.

The analysis of the skin related quality of life using the DLQI total score shows a distinct deterioration of QoL at the beginning of cycle 2 in both arms, being rather stable thereafter in the patients remaining in the observation. The absolute amount of impact on QoL according to the DLQI seems to be rather limited with about 4 out of 30 points [14]. This is due to the finding, that only the subscale “symptoms and feelings” indicated major deterioration. All other scales were hardly affected. At the time the study was designed, the DLQI seemed to be one of the most promising questionnaires to assess skin-related QoL, not least due to the data of the STEPP trial [3]. Meanwhile, a questionnaire (FACT- EGFRI-18) is available designed to assess dermatologic symptoms associated with EGFR inhibitors [15]. It should be used for future clinical trials.

In all, we were not able to find significant differences between both prophylactic treatments investigated in this trial using the NCI CTC AE v 4.0 grading system. It can be pointed out that the erythromycin arm duplicated grade 3 skin toxicity. Contrarily, by using the WoMo score we demonstrated that more patients treated with erythromycin developed moderate or severe skin toxicity at earlier time points indicating that local erythromycin should not be used as a substitute of oral tetracyclines as prophylaxis against EGFR mediated skin toxicity. We propose to use the WoMo score in addition to NCI CTCAE grading in clinical trials dedicated to study the efficacy of prophylactic or interventional measures against EGFR-mediate skin toxicity.

Moreover, rash rates are high despite prophylactic treatment. These high rates beg for further research to establish improved alternative treatment options. High expectations are placed on vitamin K for use of EGFR treatment associated skin toxicity. The prophylactic use of K1 cream was demonstrated by Ocvirk [16] and – most recently – by Schimanski et al. [17]. Regarding this, the results of the EVITA trial (NCI01345526) are eagerly awaited. EVITA is the first randomized study that evaluated the addition of vitamin K to doxycycline prophylaxis. Another promising trial (NCT03051880) investigates the preventive use of topical EGF cream. Until then the prophylactic use of tetracyclines may be regarded as a standard of care in patients undergoing anti-EGFR directed treatment.

## MATERIALS AND METHODS

### Patients

Patients with metastatic colorectal cancer were eligible to participate in the study provided they met all selection criteria listed in Table 6. No study treatment or any other procedure within the framework of the trial was performed in any patient prior to receipt of written informed consent.

**Table 6:** Inclusion and exclusion criteria

**Inclusion criteria**: Patients with wild-type RAS (KRAS and NRAS) status of metastatic colorectal cancer treatment with panitumumab according to label RAS wild-type tested in KRAS exon 2 (codons 12/13) KRAS exon 3 (codons 59/61) KRAS exon 4 (codons 117/146) NRAS exon 2 (codons 12/13) NRAS exon 3 (codons 59/61) NRAS exon 4 (codons 117/146) Treatment with pre-emptive study medication shall begin the day before treatment start with panitumumab Willingness to cope with biweekly quality of life questionnaires Written Informed consent Aged at least 18 years ECOG Performance Status 0-2 Life expectancy of at least 12 weeks Adequate haematological, hepatic, renal and metabolic function parameters: Leukocytes > 3000/mm^3^ ANC ≥ 1500/mm^3^ Platelets ≥ 100,000/mm^3^ Haemoglobin > 9 g/dl Serum creatinine ≤ 1.5 x ULN Bilirubin ≤ 1.5 x ULN GOT-GPT ≤ 2.5 x ULN (in case of liver metastases GOT / GPT ≤ 5 x ULN) AP ≤ 5 x ULN Magnesium, Calcium and potassium within normal ranges (may be substituted before study entry)
**Exclusion criteria**: Subject pregnant or breast feeding, or planning to become pregnant within 6 months after the end of treatment Subject (male or female) is not willing to use highly effective methods of contraception (per institutional standard) during treatment and for 6 months (male or female) after the end of treatment (adequate: oral contraceptives, intrauterine device or barrier method in conjunction with spermicidal jelly) Serious concurrent diseases On-treatment participation in a clinical study in the period 30 days prior to inclusion Clinically significant cardiovascular disease in (incl. myocardial infarction, unstable angina, symptomatic congestive heart failure, serious uncontrolled cardiac arrhythmia) ≤ 1 year before enrolment History of interstitial lung disease, e.g. pneumonitis or pulmonary fibrosis or evidence of interstitial lung disease on baseline chest CT scan History of HIV infection Other previous or concurrent malignancy (≤ 5 years prior to enrolment in study) except non-melanoma skin cancer or cervical carcinoma FIGO stage 0-1 if the patient is continuously disease-free Known allergic reactions on panitumumab, doxycycline or erythromycin Previous treatment with anti-cancer agents directed against EGFR (e.g. cetuximab, panitumumab, erlotinib, gefitinib, lapatinib) Skin rash existing before or due to other reasons than panitumumab treatment Other dermatologic disease that may interfere with correct grading of panitumumab induced skin rash Parallel treatment with anti-tumor agents other than panitumumab

At the time the study was initiated, patients with tumors harboring no KRAS exon 2 mutations were included to be treated according to panitumumab label. Data on the negative predictive effect of mutations beyond KRAS exon 2 (i.e. KRAS exon 3-4 and NRAS exons 2-4) were first presented during the American Society of Clinical Oncology (ASCO) Annual Meeting 2013 and published later on [18, 19]. The inclusion criteria of the current trial were changed accordingly. The amendment was approved by the ethical committee on 17^th^ January 2014.

### Study design and treatment schedule

This is a phase II, open-label, randomized, controlled, parallel-arm, multi-center study. Patients received standard tumor treatment using panitumumab in label (i.e. either as monotherapy or in combination with chemotherapy), and were randomized (1:1) to two preemptive strategies of skin toxicity prophylaxis. In order to base the trial on the best available evidence, the superior treatment arm of the phase II STEPP trial [3] was chosen as standard arm: (i) Standard arm: Skin moisturizer (in the morning on rising), sun screen (before going outdoor), doxycycline 100 mg b.i.d. starting the day before treatment start with panitumumab and for the following 8 weeks. (ii) Erythromycin arm (sequential approach): Skin moisturizer (in the morning on rising), sun screen (before going outdoor), erythromycin ointment 2% on face, hands, feet, neck, back, once daily at bedtime starting the day before treatment start with panitumumab and for the following 8 weeks; in case of skin rash ≥ grade 2 doxycycline 100 mg b.i.d. was started.

The study treatment period consisted of 8 weeks (i.e. 4 administrations of bi-weekly panitumumab).

### Study objectives

The primary objective of the study was to establish an alternative preemptive skin treatment avoiding or delaying the use of oral doxycycline in patients with wild-type (K)RAS metastatic colorectal cancer treated with panitumumab. For the primary endpoint (percentage of patients developing no skin toxicity ≥ grade 2 at any time during the first 8 weeks of treatment with panitumumab) skin toxicity was assessed using the NCI CTCAE v 4.0 criteria.

Secondary objectives comprised: (i) Evaluation of skin-related (DLQI) and global quality of life (EORTC QLQ C30); (ii) assessment of skin toxicity with a different grading scale (i.e. WoMo score).

### Study assessments

Panitumumab was administered on a bi-weekly basis according to label (i.e. cycle length was 14 days). On day 1 of every panitumumab treatment cycle the following assessments were scheduled: (i) Physical examination including weight, ECOG status and vital signs; (ii) adverse effects according the NCI CTC Version 3.0 criteria; (iii) laboratory tests including clinical chemistry and hematology; (iv) quality of life including EORTC Quality-of-life C30, skin related quality of life with DLQI; (v) skin rash assessment including NCI CTC criteria version 4.0 and WoMo scoring.

For skin toxicity grading purposes the NCI CTCAE v 4.0 was used as a primary endpoint (see Supplementary Materials). Moreover, we used the so called WoMo (Wollenberg and Moosmann) score, a thorough grading system. Briefly, the WoMo score is based on a three-part system which takes into account body involvement according to the rule of nines, percentage of facial involvement, as well as a semiquantitative description of the skin lesions by five items (erythema intensity and distribution, papulation, postulation, and scaling/crusts). The final score ranges from 0 to 100 (0 to 20 mild, 21 to 40 moderate, 41 to 100 severe acneiform eruptions) [20] (see Supplementary Materials). The assessment of skin toxicity was done by physicians trained on the NCI CTC and WoMo scores.

The assessment of overall quality of life was done using the EORTC QLQ C30. Skin-related quality of life was graded according to the dermatology quality of life index (DLQI; cf: http://sites.cardiff.ac.uk/dermatology/quality-of-life/dermatology-quality-of-life-index-dlqi/). Briefly, the DLQI consists of 10 questions to assess QoL in patients with skin disorders and can be analyzed under six subscales (symptoms and feelings, daily activities, leisure, work and school, personal relationship, and treatment). It is scored on a scale of 0 to 30; higher scores indicate more QoL impairment [21].

Tumor assessments were performed according to local practice. As all antineoplastic drugs used in this trial have a well-documented adverse event profile, the assessment of adverse events beyond skin toxicity was outside the main scope of the trial. However, serious adverse events were recorded and analyzed.

### Statistical considerations

According to data derived from the STEPP trial the development of skin toxicity grade ≥ 2 can be avoided in about 70% of patients [2]. The primary hypothesis of the current trial was that a similar efficiency could be achieved by a sequential skin treatment strategy, starting with local erythromycin administration.

Despite the shorter duration of the STEPP trial (6 weeks of treatment) its results had to be applicable to the 8 week situation in this trial because the primary endpoint in both trials was based on the worst grade skin toxicity which is reached after approximately 2 weeks of panitumumab treatment. The natural course of panitumumab induced skin toxicity after the climax at about 2 weeks is a steady recovery within the following weeks.

The statistical calculation was based on the following premises and assumptions:

According to these parameters, and using a standard single-stage phase II design by Fleming (1982), n = 37 patients evaluable for prophylactic efficacy had to be recruited. As a similar number of patients had to be recruited to the reference STEPP arm, a total number of 74 evaluable patients was required. Calculating a drop-out rate of 15% a total of 88 patients had to be included in the study.

All randomized patients who received at least one application of study medication and who had at least one post-baseline assessment of the primary endpoint variable will be the full analysis set (FAS), even if they violate the selection criteria of the study.

Skin toxicity (primary endpoint), clinical response and other rates were calculated, providing confidence intervals. In case of comparison between patient groups, these proportions were to be analyzed by Fisher´s exact test, X^2^ test or Mantel-Haenszel test (or trend test according to COCHRAN/ARMITAGE), respectively. For correlation analyses between the different quality of life and/or toxicity scores, the non-parametric test according to Spearman was preferably to be applied.

The onset of grade ≥ 2 skin toxicity was to be likewise estimated by the product limit method of Kaplan-Meier and eventually compared using the logrank test.

### Ethics

The study was approved by the Ethics Committee (EC), Medizinische Ethik-Kommission II of the Universitätsmedizin Mannheim, and all other participating institutions.

This study was conducted in agreement with the German Drug Law (AMG), ICH Harmonized Tripartite Guideline on Good Clinical Practice, the „Verordnung über die Anwendung der Guten Klinischen Praxis bei der Durchführung von klinischen Prüfungen mit Arzneimitteln zur Anwendung am Menschen“ [22] and the Declaration of Helsinki (Tokyo, Venice, Hong Kong, Somerset West and Edinburgh amendments) [23].

## SUPPLEMENTARY MATERIALS


