# Supplementary material for: AIO LQ-0110: a randomized phase II trial comparing oral doxycycline versus local administration of erythromycin as preemptive treatment strategies of panitumumab-mediated skin toxicity in patients with metastatic colorectal cancer
Source: Oncotarget. 2017 Sep 23;8(62):105061–71. doi: 10.18632/oncotarget.21249 (PMC5739620; doi:10.18632/oncotarget.21249)
Supplement: Supplementary file 1 [file oncotarget-08-105061-s001.pdf]

AIO LQ-0110: a randomized phase II trial comparing oral doxycycline versus local administration of erythromycin as preemptive treatment strategies of panitumumab-mediated skin toxicity in patients with metastatic colorectal cancer

SUPPLEMENTARY MATERIALS

Panitumumab dose reduction due to skin toxicity, by patient

Dose reduction	Doxycycline	Erythromycin	Total
N	*41*	*39*	*80*
no	41 (100%)	38 (97%)	79 (99%)
yes	-	1 (3%)	1 (1%)

Panitumumab dose delay, by patient

Dose delay	Doxycycline	Erythromycin	Total
N	*41*	*39*	*80*
no	39 (95%)	38 (97%)	77 (96%)
yes	2 (5%)	1 (3%)	3 (4%)

Skin toxicity grading scale according to NCI CTCAE v 4.0 (see also: https://evs.nci.nih.gov/ftp1/CTCAE/CTCAE_4.03_2010-06-14_QuickReference_8.5x11.pdf)

Adverse event	Grade				
	1	2	3	4	5
Rash acneiform	Papules and/or pustules covering ≤ 10% BSA, which may or may not be associated with symptoms of pruritus or tenderness	Papules and/or pustules covering 10 - 30% BSA, which may or may not be associated with symptoms of pruritus or tenderness; associated with psychosocial impact; limiting instrumental ADL	Papules and/or pustules covering > 30% BSA, which may or may not be associated with symptoms of pruritus or tenderness; limiting self care ADL; associated with local superinfection with oral antibiotics indicated	Papules and/or pustules covering any % BSA, which may or may not be associated with symptoms of pruritus or tenderness and are associated with extensive superinfection with IV antibiotics indicated; life-threatening consequences	Death

Definition: A disorder characterized by an eruption of papules and pustules, typically appearing in face, scalp, upper chest and back.

ADL: Activities of daily living.

Skin toxicity grading score according to Wollenberg and Moosmann (WoMo score)

	Value (intermediate values such a 0.5, 1.5 and 2.5 are allowed)
(1) Colour intensity of erythema within the borders of the affected area (none = 0, pale red = 1, moderate = 2, dark red = 3)	
(2) Erythema distribution density within the borders of the affected area (none = 0, scattered = 1, intermediate = 2, dense = 3)	
(3) Papulation (number, size, and discolouration with a balanced score of 0-3)	
(4) Pustulation (number, size, and discolouration with a balanced score of 0-3)	
(5) Scaling/Crust formation (intensity, size, and spread with a balanced score of 0-3)	
C = Sum (1-5)	
A = Body involvement (extent of body lesion, 1- 100%, according to the rule of nines)	
B = Facial involvement (extent of lesion in the face, 0-100%)	
Final score (1/4 A + 1/4 B + 10/3 C)	

Grading by final score: 0 – 20: mild; 21 – 40: moderate, 41 – 100: severe).
